# Acknowledgment to Reviewers of *Entropy* in 2021

**DOI:** 10.3390/e24020217

**Published:** 2022-01-29

**Authors:** Entropy Editorial Office

**Affiliations:** MDPI AG, St. Alban-Anlage 66, 4052 Basel, Switzerland

Rigorous peer-reviews are the basis of high-quality academic publishing. Thanks to the great efforts of our reviewers, *Entropy* was able to maintain its standards for the high quality of its published papers. Thanks to the contribution of our reviewers, in 2021, the median time to first decision was 18 days and the median time to publication was 39 days. The editors would like to extend their gratitude and recognition to the following reviewers for their precious time and dedication, regardless of whether the papers they reviewed were finally published:
Abah, ObinnaKyzas, GeorgeAbbas-Aghababazadeh, FarnooshLa Cour, BrianAbbaspour, SaraLadaru, RalucaAbbot, Dorian SchuylerLadyman, JamesAbbruzzo, AntoninoLages, JoséAbdel Razek, Ahmed Abdel KhalekLaguna, HumbertoAbdel-Aty, Abdel-HaleemLai, MirkoAbdel-Aty, MahmoudLain, Jenn-KaieAbdel-Latif, AhmedLakner, ZoltanAbdelsalam, Sara I.Lamb, Don C.Abdi, AsadLamberti, Pedro WalterAbdulrahman, HasanLambert-Mogiliansky, ArianeAbebe, AmareLambiase, GaetanoAbel, MarkusLandi, Gabriel T.Abellán, JoaquínLangmann, EdwinAbid, AdnanLanillos, PabloAbodayeh, KamalLansky, JanAbouelregal, Ahmed E.Lanusse, PatrickAbouenour, LahsenLanzetta, FrancoisAbundo, MarioLarralde, HernanAbyzov, AlexanderLarson, JonasAcevedo, Rubén CarlosLarsson, RolfAcharya, JayadevLatif, Muhammad AmerAcosta, Antonio JoséLaudisa, FedericoAcosta-Avalos, DanielLauricella, MarcoAdachi, KoheiLaurini, Márcio PolettiAdali, SarpLavanga, MarioAdami, ChristophLawrence, JayAdamic, PeterLazaroiu, GeorgeAdeyemi-Ejeye, FemiLech, PiotrAdlam, EmilyLee Ho, LindaAdriaans, Pieter W.Lee, AndrewAgbo, Friday JosephLee, Hyung-TaeAgüero-Chapin, GuillerminLee, InAguiar, MarcusLee, Jae WooAguilar, Jean-PhilippeLee, JaesungAgus, MirianLee, JeonAhmad, JawadLee, Jeong-Eun (Kate)Ahmad, RaufLee, KyeongjunAhn, JoonLee, Kyu-haengAiazzi, BrunoLee, Sang HoonAiordachioaie, DorelLee, Shyi-LongAissa, Mohamed HassanineLee, Tian-FuAjates, RaquelLee, WoojooAkamatsu, NaokiLefèvre, EricAkhshani, AfshinLehikoinen, AnnukkaAkhtar, ZahidLeinonen, MarkusAkinyemi, LanreLeithardt, Valderi Reis QuietinhoAkram, SheerazLeMay, Stephen ArthurAlam, Miah Md AshrafulLemnaru, CameliaAl-Amri, MohammadLenk, PeterAlbantakis, LarissaLentmaier, MichaelAlbuquerque, Daniel FilipeLenzi, Ervin KaminskiAlcantud, José CarlosLeón, AlejandroAlDajani, Phil Iyad MuhsenLeonardi, GiuseppeAldaya, IvanLerner, PeterAlderete, C. HuertaLevina, AllaAledo, Juan A.Levitan, JacobAlevtina, GostevaLew, SergioAlexakis, HarisLey, ChristopheAlexey, KanelLeyvraz, FrancoisAlexeyev, StanislavLi, BaokuanAlfinito, EleonoraLi, ChengqingAli, AkramLi, Chia-JungAliyu, AliyuLi, GuanchenAlkhnbashi, Omer S.Li, JianningAllani, SabriLi, JieAlonso, Joan F.Li, PengAlquier, PierreLi, ShaoAl-Rifaie, Mohammad MajidLi, XiangAlsharif, Mohammed H.Li, Xiao-SenAltschul, Brett D.Liang, Chih-ChinAlvaredo, PaulaLiao, HaoAlvarez-Lacalle, EnricLiao, LingminAlvarez-Meza, A. M.Licitra, GaetanoAlves, Gleifer VazLiechti, FelixAly Abdallah, Abdelraheem MahmoudLim, Dong-KukAlzabut, JehadLim, JongBeomAman, HaroonLima, Leonardo dos SantosAmaral, Marcelo M.Lin, Che-TsungAmero, CarlosLin, Chin-FengAmigó, José MaríaLin, ChuandongAmosov, GrigoriLin, Feng-ChengAnagnostis, AthanasiosLin, Hua-YiAnagnostopoulos, Nikolaos AthanasiosLin, JasonAndaluz, JoaquínLin, Jia-WenAndersen, HollyLin, Jun-LinAndersson, Sean B.Lin, SijinAndrade, AlexandreLindsey, Kathryn A.Andretta, MassimoLiška, MarekAndriyanov, NikitaLittle, Douglas J.Andronikos, TheodoreLittle, Roderick J. A.Angeles, FelipeLiu, Guan-ZhengAngelico, RuggeroLiu, HonghuAnghel, Dragos-VictorLiu, KefeiAnglin, JamesLiu, ShuangzheAnselm, BlumerLiu, WeiboAntonelli, LauraLiu, ZhengminAntonio, Stella FabioLizcano Casas, DavidAntunes, MarceloLlorens, Alfonso SuárezAntunes, MárioLloret, JaimeAnysz, HubertLobo, Iarley PereiraAono, YoshinoriLocatelli, EmanueleApostolova, ElenaLomba, EnriqueAppadu, Appanah RaoLombardi, AngelaAprea, CiroLombardi, OlimpiaArafat, HishamLong, Gui-LuAraujo, AlvaroLongobardi, MariaArauzo, A.Lonkwic, PawełArdenghi, Juan SebastiánLoos, SarahArgyrakis, PanosLoperfido, NicolaAricò, PietroLópez Jiménez, Petra AmparoAristov, Vladimir V.López Pajares, DiegoAriza-Villaverde, Ana BelénLopez Paniagua, IgnacioArizmendi, Constancio MiguelLopez, Juan M.Arnerić, JosipLópez-Mancilla, DidierAros, RodrigoLopez-Martin, ManuelArraut, IvanLorentzou, SouzanaArroyo, JesúsLosada, Juan CarlosArroyo-Figueroa, GustavoLouhenkilpi, SeppoArsac, Laurent M.Lovričević, NedaArslan-Ayaydin, ÖzgürLu, Chi-KenArtemiou, AndreasLu, ZhizhongArumí, DanielLuca, AdrianAsaad, SabaLuckner, MarcinAsci, FrancescoŁudzik, KatarzynaAscione, GiacomoLughofer, EdwinAshrafuzzaman, MohammadLuk, RobertAsim, MuhammadLuo, Ming-XingAslam, MuhammadLuongo, OrlandoAsllani, MalborLupo, CosmoAsselineau, Charles-AlexisLuque, BartolomeAstolfi, DavideLutsko, James F.Astorino, MarcoM′zoughi, FaresAszalós, LászlóMacci, ClaudioAtanackovic, TeodorMacedo, PedroAtinafu, Dimberu GeremewMachado, IvanAugust, EliasMachuca, Juan L. MataAur, DorianMachura, LukaszAurelian, IsarMaciel, CarlosAusloos, MarcelMacris, NicolasAvci, MustafaMacura, DraganaAvdi, EvrinomyMadau, Fabio A.Avino, PasqualeMadouasse, AurélienAwad, Mohamed M.Mady, Carlos Eduardo KeutenedjianAwadallah, AymanMafessoni, FabrizioAzam, Naveed AhmedMagafas, LykourgosAzar, Ahmad TaherMaggi, MarioAzhagiya Singam, Ettayapuram RamaprasadMah, MatthewAziz, FurqanMaheshwari, AdityaBaccala, Luiz AntonioMahmoud, Gamal M.Badihi, HamedMairhofer, LukasBadulescu, AlinaMaisto, DomenicoBaek, ChangryongMaiti, MoinakBagnoli, FrancoMaity, DipankarBaiesi, MarcoMajewska, ElżbietaBailón, RaquelMajewski, MaciejBajdor, PaulaMajidi Nezhad, MeysamBajic, DraganaMajka, MichalBak-Coleman, JosephMajor, SethBakhtin, VictorMajtey, Ana P.Balado Frias, JesusMakowiec, DanutaBalasco, MariannaMakowski, MarcinBalaz, AntunMakris, ChristosBalicki, JerzyMalara, FrancescoBallani, FelixMałecki, KrzysztofBallesteros, Dora M.Malkin, MikhailBallmann, ChristopherMallatt, JonBallo, AndreaMalo, JesusBamba, KazuharuMalutan, RaulBan, MasashiManis, GeorgeBancal, Jean-DanielMan′ko, MargaritaBanerjee, ImonMan′ko, OlgaBania, PiotrManko, Vladimir I.Bansal, KanikaManolakos, DimitrisBarabino, BenedettoManolescu, AndreiBarai, ParamitaMantzaris, Alexander V.Baranowski, JerzyManzano Paule, GonzaloBarbier, JeanManzano, GonzaloBarbosa, Anderson L. R.Maragakis, MichaelBarbu, Vlad StefanMaraj, ArianitBaric, DanijelaMarateb, Hamid RezaBarinova, SophiaMarcolli, MatildeBariviera, Aurelio FernándezMarczyk, MichalBarmparis, Georgios D.Mares, IleanaBarnett, LionelMarijuán, PedroBarquero-Pérez, ÓscarMarinello, FrancescoBarra, FelipeMarini, MarcoBarranco-Chamorro, InmaculadaMarinov, PenchoBas, JoanMarkatou, MarianthiBasaran, CemalMarkoli, BoštjanBasilio, MarcioMarkov, KonstantinBasu, BidrohaMarković, DimitrijeBatchuluun, GanbayarMarle, Charles-MichelBatista, MarcoMarmo, GiuseppeBauer, DietmarMarmur, AbrahamBaumann, Michael HeinrichMarsili, StefaniaBausys, RomualdasMarszk, AdamBautu, ElenaMartian, AlexandruBayat, AbolfazlMartín-de León, JudithBeare, Brendan K.Martínez García, MiguelBechhoefer, EricMartinez Prata, DiegoBecker, AlexanderMartínez Torres, JavierBeims, Marcus W.Martinez, Alexandre SoutoBekker, AndrietteMartinez-Graullera, ÓscarBel, GolanMartinez-Val, Jose M.Bełdycka-Bórawska, AnetaMartino, AlessioBeleznai, CsabaMartino, Tartaglia GianlucaBellingeri, MicheleMartins, AndréBellomo, NicolaMartyushev, LeonidBelogolovskii, MikhailMary, PhilippeBelyaev, EvgenyMarzec-Schmidt, KatarzynaBenato, AlbertoMarzen, SarahBenatti, FabioMarzi, ClaudiaBenavente, JuanaMasegosa, Andres R.Benenti, GiulianoMasoliver, JaumeBenioff, PaulMassart, EstelleBenito, Rosa M.Masuda, ShumpeiBen-Naim, AriehMateljevic, MiodragBentes, Sónia R.Mateo, Jesús MartínezBerchialla, PaolaMatera, Juan MauricioBerdichevsky, Daniel BenjaminMateus, PauloBereyhi, AliMathelin, LionelBereziński, PrzemysławMatilla-García, MarianoBergland, OlvarMatsoukas, ThemisBeris, AntonyMatsumoto, RyutarohBernardes, Nadja KolbMatsuno, KenBernroider, GustavMatsuno, KoichiroBersimis, SotirisMatt, Carlos Frederico TrottaBeskopylny, AlexeyMattas, KonstantinosBetta, MonicaMattingly, HenryBettelheim, EldadMaturi, EileenBhattacharya, DevanjanMatuszak, MagdalenaBhowmik, DebsindhuMatutinović, IgorBhuiyan, AmranMatzkin, AlexBialynicki-Birula, IwoMayer, ChristopherBian, XinMayo-Iscar, AgustinBielecka, MarzenaMayor, JesusBier, MartinMaziero, JonasBilancia, PietroMazurek, MiroslawBilén, Sven G.McArthur, DavidBimbo, FrancescoMcCabe, BrendanBinder, PhilippeMcDanel, BradBioglio, LivioMcGuire, MichaelBiscarini, FilippoMd Borhan, MiaBishop, A. R.Mediano, Pedro A. M.Bisker, GiliMedina, Jose M.Biurrun, JoseMei, GiampieroBizarro, João P. S.Meisen, TobiasBjörn, SchenkeMeisenheimer, PeterBłasiak, PrzemysławMejía, AndrésBlecich, PaoloMejia-Parra, DanielBlin, AlexMel, RiccardoBlokhin, AndreyMelbourne, JamesBlythe, RichardMelgarejo, MiguelBocharova, Irina E.Melichar, HeatherBódai, TamásMelkikh, Alexey V.Bodini, AntonellaMellin, PatriciaBoivin, PierreMellor, JosephBokde, NeerajMelnikoff, DavidBolancé, CatalinaMelnikov, AlexanderBolanowski, MarekMelnikov, Alexey A.Bolonek-Lasoń, KatarzynaMelnychenko, OleksandrBomantara, RadityMendes, JoséBonanno, ClaudioMendez-Bermudez, J. A.Bonchev, DanailMenendez, Hector D.Bonilla Licea, DanielMenezes, TelmoBonometto, Silvio A.Merlano, JuanBonomo, FlaviaMernik, MarjanBonopera, MarcoMeron, EhudBonora, LorianoMescia, LucianoBopp, Fritz W.Mesfioui, MhamedBordag, MichaelMesjasz, CzesławBordel Sánchez, BorjaMessina, EleonoraBorges, Ernesto P.Messina, FrancescoBormashenko, EdwardMezei, JózsefBorondo, FlorentinoMian, AmmarBorowska, MartaMichaelian, KaroBosa, SilviaMichailidis, Panagiotis D.Bossé, ÉloiMichiardi, PietroBosse, JensMielniczuk, JanBouffanais, RolandMigallón, HéctorBoutayeb, HalimMigilinskas, DariusBovino, Fabio AntonioMihaescu, Cristian MarianBoyvalenkov, PeterMihai, AdelaBranciard, CyrilMihail, MeganBrandt, Steven R.Mihalcea, Bogdan VasileBranicki, MichalMihaljevic, MiodragBrasic, JamesMiholca, DianaBratianu, ConstantinMikhailov, EvgenyBraun, ChristophMikhailovich, Sergey VasinBretti, GabriellaMikki, SaidBretto, AlainMilchev, AndreyBrito, FredericoMilenković, MilošBriz-Redón, ÁlvaroMilic, Dominika CrnjacBroadbridge, PhilipMillen, JamesBroniatowski, MichelMiller, BorisBross, Shraga I.Mimoso, José P.Brotons, Jose M.Mimura, KazushiBrowne, DanMinaee, ShervinBrunelli, MatteoMiniero, Daniela ValeriaBruni, RenatoMirea, LetitiaBrzdek, JanuszMiret-Artés, SalvadorBub, JeffreyMirkes, Evgeny M.Budini, Adrián A.Mironowicz, PiotrBueno, PabloMirza, Mohammad MerajBuhse, ThomasMirzakhalili, EhsanBuick, JamesMiskiewicz, JanuszBukovsky, IvoMisra, AvijitBulinski, AlexanderMiszczak, JarekBull, JamesMitianoudis, NikolaosBulnes, FranciscoMitroi-Symeonidis, Flavia-CorinaBurattini, LauraMitrović Dankulov, MarijaBureika, GintautasMitsukura, YasueBuscarino, ArturoMiura, KenjiroBusemeyer, JeromeMjahed, MostafaBustos-Marún, RaúlMnasri, SamiBusu, MihailMobayen, SalehBuszko, MichalMöbus, ClausButurac, GoranMoca, Catalin PascuButusov, DenisMocenni, ChiaraByeon, HaewonMöckl, LeonhardBykhovsky, DimaMogeritsch, JohannCaban, JacekMoghimi, M. A.Caceres, Manuel OsvaldoMohammad, AhmadCaesarendra, WahyuMohd Noor, Noor FadiyaCai, Rong-GenMol, Jan A.Cai, YapingMoldovan, DarieCaicedo, AlexanderMoldoveanu, FloricaCajas García, Juan CarlosMolina, M. DoloresCalatroni, AlbertoMon, Yi-JenCalcagnile, LucioMoneta, AlessioCaldarola, FabioMonge, Rodrigo GomezCalderaro, LucaMonkewitz, Peter A.Calderisi, MarcoMonroy, RaúlCaliva, FrancescoMonterde, JuanCalloni, EnricoMontero, MiquelCalvo-Pardo, HectorMontes, Maria JoséCalzetta, EstebanMontévil, MaëlCámara Chávez, GuillermoMontoya, FaustoCambon, ClaudeMontoya, Ivan AlonsoCaminiti, GiuseppeMontoya, OscarCamiola, Vito DarioMoon, Kevin R.Camp, Jeremy V.Moon, KyuminCampajola, CarloMorabito, Francesco CarloCampbell, HarlanMoral-García, SerafínCampisi, MicheleMoraru, LuminiţaCampo, Adrià TausteMorawetz, KlausCampos Cantón, EricMoreira-Filho, Carlos AlbertoCandeloro, LucaMoreno, EliasCannata, GiovanniMoreno, Emma MuñozCano, AndrésMoreno-Ramírez, Luis MiguelCao, BingyangMoreo Fernández, AlejandroCaprara, SergioMoret-Tatay, CarmenCaprini, LorenzoMorison, GordonCaraiani, PetreMorita, SatoruCarbone, AnnaMorla, RicardoCardenas-Juarez, MarcoMorozov, Sergey PavlovichCarlo, GabrielMorrison, RebeccaCarlson, CharlesMoruzzi, Rodrigo BragaCarmi, AvishyMoshou, AlexandraCarpena, PedroMoskalets, MichaelCarrasco, MiguelMotamed, CyrusCarrillo-Pedroza, Francisco R.Motegi, KaijiCarvalho, Luiz MaxMoulos, PanagiotisCasali, Adenauer GirardiMoyano, LuisCasarin, RobertoMoysis, LazarosCastellano, ClaudioMuga, GonzaloCastellanos-Dominguez, GermanMuhammad, Mannan SaeedCastelli, FabrizioMühlhaus, TimoCastiglione, ArcangeloMukhamedov, Farrukh MaksutovichCastiglioni, PaoloMukhametzhanov, MaratCastillo, OscarMukherjee, ManasCastro, EnriqueMuldoon, ConorCastro, HenriqueMuñoz, EnriqueÇatak, EvrenMurari, AndreaCatalina, MirelaMurcio-Villanueva, RobertoCaţaron, AngelMurguía, J. S.Cauet, SebastienMurillo-Escobar, Miguel AngelCauteruccio, FrancescoMurta Junior, Luiz OtávioCazelles, ElsaMusek, JanekCekirge, Huseyin MuratMusio, MonicaCelano, UmbertoMuthugala, VirajCelikkanat, HandeMuthuswamy, BharathwajCeolin, AndreaMylnikov, LeonidČereška, AudriusNabajeet, BarmanČerničková, IvonaNabity, JamesCetto, Ana MariaNagaraj, NithinChai, ZhenhuaNagashima, KengoChamberlin, Ralph V.Nagata, KojiChamodrakas, IoannisNagy, AgnesChan, RosaNakamura, AtsushiChandra, DaryusNakamura, IsseiChang, Cheng-Hsun-TonyNakamura, KensukeChang, Chih-WenNakamura, MorikazuChang, Ching-LuehNakata, YoshifumiChang, Pei-ZenNakonieczny, ŁukaszChang, ZheNalepa, JakubChantasri, AreeyaNalmpantis, DimitriosCharakopoulos, AvraamNambu, YasusadaCharvat, JakubNapoli, ChristianChatterjee, AtanuNaqvi, Rizwan AliChaves, Daniel A. R.Naraei, ParisaChechkin, OleksiiNarayanan, AjitChehri, AbdellahNaro, AntoninoChella, AntonioNassar, MazenChen, Chin-LingNavarro-Mesa, Juan L.Chen, EnguoNazarkevych, MariiaChen, HongtianNazarov, Dmitry MikhailovichChen, Hong-YiNechita, ElenaChen, JiahongNehaniv, ChrystopherChen, JiaweiNejadsattari, FarshadChen, JinghuiNekrashevych, VolodymyrChen, JunNemeth, KarolyChen, Jyh-ChengNeves, António J. R.Chen, Ming-ChengNewson, AlasdairChen, ShiNguyen, BenjaminChen, Yi-ChungNguyen, HoangCheng, Chi-HoNguyen, Phan-MinhCheng, Shyi-ChyNguyen, Tuan AnhCheong, Kang HaoNicolaou, NicolettaChernov, AndreyNicolis, OriettaCherstvy, AndreyNie, DongChesneau, ChristopheNielsen, HeberChevallier, JulienNiemczyk, JerzyChiatti, LeonardoNigmatullin, RaoulChica, Jose-Maria SerranoNijkamp, JasperChih, MingchangNikolaevich Sapunov, ValentinChillali, AbdelhakimNikolic, HrvojeChinnici, GaetanoNikolopoulos, GeorgiosChiu, Yi-BinNikpour, ArminChmelik, FrantisekNikulchev, EvgeniyCho, DongilNikulov, Aleksey V.Choi, HeeyoulNiven, RobertChoi, Jeong RyeolNobile, Marco S.Choi, JunesangNoce, CanioChotorlishvili, LevanNoguchi, YoshihiroChou, Pei-TingNouioua, MouradChoudhuri, IndraniNovaes, MarcelChowdhury, SouravNovikova, EvgeniaChristodoulakis, JohnNowosad, JakubChristov, Ivan P.Nunes, AbrahamChruscinski, DariuszNúñez Cobo, JorgeChuang, Cheng-HungNyka, KrzysztofChuang, Hung-YiOaksford, MikeChung, Kung-MingObi, ShinnosukeChung, KyungyongObradovic, RatkoCiccarello, FrancescoObukhov, Artyom DmitrievichCiccioli, AndreaOchoa, AlbertoCicirello, VincentOcker, Gabriel KochCieplak, MarekOdeh, Suhail M.Cieśliński, JanOdloak, DarciCilluffo, DarioOggier, FrederiqueCinelli, MatteoOh, BeomseokCinti, FabioOhsawa, YukioCirugeda, Eva MaríaOkabe, YutakaCiuonzo, DomenicoOkopinska, AnnaCivera, MarcoOkorokov, VitalyCivit-Balcells, AntónOlbrich, EckehardClairambault, JeanOlbryś, JoannaCleaver, GeraldOldofredi, AndreaClemence, Dominic P.Olivares, FelipeClemente, Filipe ManuelOlivares-Robles, Miguel AngelClerc, MarcelOliveira, Arlindo L.Coco, IginoOliveri, RobertoCodetta Raiteri, DanieleOmlin, Christian W.Cohen, AsafOmotayo, AbiodunCohen, GilOncioiu, Ionica HolbanCollazo, JoaquínOng, Chi WeiCome, EtienneOnken, ArnoComes, Calin-AdrianOno, KanjiCondurache, DanielOppel, MarkusCongedo, Paolo MariaOprea, IulianaConradt, ReinhardOrabona, FrancescoConsole, RodolfoOrganisciak, PeterConstantin, Cristinel PetrisorOrorbia, Alexander G.Contreras-Reyes, Javier E.Oros, Georgia IrinaConvertino, MatteoOrtale, RiccardoCooke, JamesOrtiz-Medina, JosueCorda, ChristianOrzack, StevenCorduas, MarcellaOsakue, Edward E.Coronel, AnibalO′Shaughnessy, DouglasCorso, GilbertoOshita, NaritakaCortes-Huerto, RobinsonOsińska, MagdalenaCorti, David S.Osiurak, FrançoisCosma, AntonioÓskarsdóttir, MaríaCosta, José A. F.Oskay, Rıza GörkemCosta, Max Henrique MachadoOsmolovskii, Nikolai P.Costinas, SorinaØstergaard, JanCotfas, Liviu-AdrianOszust, MariuszCousineau, DenisOtani, TomohiroCraus, MiticaOttaviani, CarloCravero, CarloOtto, Daniel P.Cremaschini, ClaudioOu, CongjieCreutz, MathiasOuimet, FrédéricCrippa, PaoloØygarden, MortenCruzeiro, Emmanuel ZambriniOzawa, MisakiCruz-Reyes, LauraOzel, OmurCsató, LehelOzkaynak, FatihCsernak, GaborPadilla, PabloCui, FangdaPaes, AlineCui, HuijuanPáez-Hernández, RicardoCullen, MaellPaggi, HoracioCurmi, Paul M. G.Pagnini, GianniCushman-Roisin, BenoitPagnottoni, PaoloCusido, JordiPaige, RobCybulski, GerardPaillusson, FabienCyganek, BogusławPal, Priyo ShankarCyklis, PiotrPalagyi, KalmanCyril, ElouardPalazzo, PierfrancescoCzerwiński, ArturPaleologu, ConstantinD′Amours, ClaudePalestini, ArsenDa Costa, Bruno GomesPalma, Gioacchino MassimoDa Lio, BeatricePanagiotakis, CostasDa Luz, Marcos Gomes EleutérioPancioni, LucaDa Mota Vilela, André LuisPandey, RajeshDa Silva Baptista, MuriloPankratova, EvgeniyaDa Silva, Eduardo CostaPannone, MarilenaD′Abramo, GermanoPantaleo, EsterDąbrowska, JolantaPapadogiannis, DimitriosDaffertshofer, AndreasPapadopoulou, MariaDaga, Alessandro PaoloPapana, AngelikiDahash, AbdulrahmanPapini, GiorgioDahoo, PierrePapousek, IlonaDahyot, RozennPappalardo, GiuseppinaDairi, AbdelkaderPappas, NikolaosDajka, JerzyPappas, SteliosDamaševičius, RobertasParada, RaúlDamer, BruceParaoanu, SorinDamián-Ascencio, César EduardoParaskevas, MichaelD′Amico, GuglielmoPardo Martínez, Clara InésD′Andreagiovanni, FabioParinov, Ivan A.Daneshazarian, RezaParisi, RaffaeleDarmon, DavidParisio, FernandoDashtipour, KiaPark, ChanyoungDaskalopoulou, IrenePark, Cheol YoungDassios, AngelosPark, ChoonkilDassios, IoannisPark, HanhoonDasygenis, MinasPark, HerieDavidson, RusselPark, Jeong-SooDavidy, AlonPark, JihongDavydenko, YevhenPark, JinhongDawid, PhilipPark, JinwooDe Alencar, Marcelo SampaioPark, SangunDe Andrés-Sánchez, JorgeParra Arévalo, María IsabelDe Araújo, João M.Parte, LauraDe Bragança Pereira, Carlos AlbertoPasten, DenisseDe Brevern, Alexandre G.Pastor, Juan ManuelDe Castro, MarioPastor-Escuredo, DavidDe J. León Montiel, RobertoPastuszak, GrzegorzDe la Calle, AlbertoPaszkiewicz, AndrzejDe la Fraga, Luis G.Patanè, LucaDe la Fuente-Mella, HannsPatel, Ravi G.De la Pena, Lisandro HernandezPatikova, ZuzanaDe la Sen, ManuelPatino, JoseDe la Torre-Montero, Julio C.Patrascioiu, CristianDe Leon, ManuelPatriarca, MarcoDe Martino, IvanPatwardhan, Amol S.De Menezes, Marcio ArgolloPaulus, DietrichDe Oliveira Júnior, SilvioPaunkovic, NikolaDe Oliveira, Julio CesarPavaloaia, Vasile DanielDe Oliveira, Marcos CesarPavelka, MichalDe Oliveira, Mário JoséPavol, PriputenDe Oliveira, Roberto Celio LimaoPawar, ShrikantDe Paolis, FrancescoPaweł, DymoraDe Paz Carmona, HectorPawlicki, MarekDe Sanctis, Angela AnnaPawłowski, MarcinDe Sanctis, MauroPawłowski, PawełDe Souza, Camila P. E.Pazira, HassanDe Vito, SaverioPchelintsev, AlexanderDe Vries, BertPeixoto de Faria, José GeraldoDebiossac, MaximePejic Bach, MirjanaDebono, Carl JamesPeleato, BorjaDębowski, ŁukaszPelta, DavidDedecius, KamilPeña, Francisco J.DeDeo, SimonPena, Rodrigo F. O.Dedkov, Georgii V.Pennini, FlaviaDedu, SilviaPepe, Francesco V.Deffner, SebastianPepperell, RobertDegollado, Juan CarlosPerak, BenediktDeguchi, TetsuoPerarnau-Llobet, MartíDel Pino, JavierPereira, Éder J. A. L.Del Rio Amador, LeninPereira, IsabelDelcampo, AdolfoPerez Alday, Erick AndresDelcea, CameliaPérez, GustavoDelibra, GiovanniPeris, AlfredDelrieux, ClaudioPerkins, WillDemetrescu, IoanaPerng, Cherng-tiaoDemidenko, EugenePerotto, Humberto L.Dénes, AttilaPerpetuini, DavidDeng, Lih-YuanPescapè, AntonioDeniziak, StanisławPesta, MichalDerbez, PatrickPetcu, DanaDerkach, IvanPetcu, Monica AurelianaDesmet, BernardPetiziol, FrancescoDessalles, Jean-LouisPetrović, AndrijaDeufemia, VincenzoPetrović, MilicaDeuszkiewicz, PiotrPettini, MarcoDeveci, MuhammetPham, Viet-ThanhDey, RounakPiangerelli, MarcoDi Digioacchino, DanielePierannunzio, DanielaDi Gregorio, SalvatorePietrowicz, SławomirDi Marco, RobertoPikovsky, ArkadyDi Molfetta, GiuseppePiltan, FarzinDi Prisco, AliciaPinčák, RichardDi Zio, SimonePinchas, MonikaDiana, GiovanniPineda, MiguelDíaz Ferreyra, Nicolás E.Pinheiro, MarioDíaz, JoséPinho, Armando J.Díaz, María GarcíaPinto, Armando N.Diaz, Rocio GonzalezPipień, MateuszDíaz-Castillo, CarlosPirandola, StefanoDíaz-Díaz, NorbertoPirogov, SergeyDicandia, Francesco AlessioPiskorski, JaroslawDiego, IsidroPisla, DoinaDieks, DennisPittino, FedericoDiep, Hung T.Piwowarski, MarianDíez, Luis FranciscoPizarro, Mario MartínezDimililer, KamilPlastina, FrancescoDimitriadis, PanayiotisPlastino, Angel RicardoDimitriu, Dan-GheorghePlastino, AngeloDing, JintaiPlatiša, Mirjana M.Ding, NiPleimling, MichelDiop, SettePlotnitsky, ArkadyDipnall, JoannaPoderini, DavideDiraco, GiovanniPodgorelec, BlažDixon, David A.Podobnik, BorisDixon, JamesPolanski, ArnoldDiykh, MohammedPołap, DawidDjakovic, DamirPoletti, DarioDjeziri, MohandPoljak, IgorDobeš, JosefPons, MiquelDobrokhotov, SergeiPonta, LindaDolganov, Pavel VladimirovichPopa, Călin-AdrianDolgy, Dmitry V.Popa, Gabriel NicolaeD′Oliveira, Rafael Gregorio LucasPopkov, Yuri S.Doll, KlausPorta, AlbertoDöller, MarioPorto Pato, MauricioDomanski, TadeuszPortugal, RenatoDomb, MenachemPorwik, PiotrDomínguez, DanielPoshtkohi, AlirezaDomino, KrzysztofPostma, EricDondi, PiercarloPotapov, IlyaDong, RuifengPoulose, AlwinDonner, ReikPoveda-Cuevas, Freddy JacksonDonzé, LaurentPradeep Ram, Angia SriramDotov, DobromirPrados, AntonioDoulamis, AnastasiosPrado-Velasco, ManuelDoulamis, NikolaosPrati, RonaldoDowding, IrenePratt, ScottDowlatshahi, Mohammad BagherPrause, ChristianDragicevic, Arnaud Z.Preda, VasileDragisa, StanujkicPrendergast, LukeDrago, CarloPrentner, RobertDrakopoulos, GeorgiosPrestipino, SantiDresp-Langley, BirgittaPreviato, EmmaDritsaki, MelinaPrice, HuwDu, JingshanPrieto Guerrero, AlfonsoDuan, QiangPrieto, Jesús-IgnacioDuda, JerzyPrieto-Castrillo, FranciscoDuda, PiotrPrikhodko, IgorDudin, AlexanderPripp, Are HugoDufour, GabrielProbierz, BarbaraDulău, MirceaProesmans, KarelDumitrascu, GheorgheProkopenko, MikhailDumitru, Cristian-DragoşPrömel, DavidĎurica, MarekPross, AddyDzemyda, GintautasProsviryakov, EvgeniiDzikuć, MaciejProtzner, Andrea B.Efremov, Maxim A.Providas, EfthimiosEftimie, RalucaPryadko, Leonid P.Egger, JanPrzybyła-Kasperek, MałgorzataEhlers, Ricardo SandesPuertas-Centeno, DavidEiras Fernández, JesúsPullen, Keith RobertEisencraft, MarcioPurcar, MariusEl Alaoui, AhmedPutzu, LorenzoEleuch, HichemPyshkin, PavloEl-Hadedy, MohamedQi, GuanqiuEl-Khazali, ReyadQu, ChuangEl-Morshedy, MahmoudQuapp, WolfgangEl-Qady, Gad M.Quax, RickEl-Sayed, AhmedQueirós, SandroElsharkawy, MohsenQuevedo, DanielElze, ThomasQyyum, Muhammad AbdulEnders, PeterRadescu, RaduEndo, HiroyukiRadhakrishnan, SrinivasanEnea, MarcoRadovic, MirjanaEngland, JeremyRae, AlastairEo, Soo HyungRafaralahy, HuguesEremenko, SergeiRagusa, AntonellaEremeyev, VictorRagusa, Maria AlessandraEriksson, Rikard H.Rahman, Mohammad MansurEscamilla Herrera, Lenin FranciscoRahmani, RaminEsmaiel, HamadaRahmat, AminEspadinha-Cruz, PedroRahmati, MehdiEsquivel, Rodolfo O.Raimondi, GianfrancoEstellé, PatriceRaimondo, FedericoEvangelista, Luiz RobertoRaj, NawinEvans, MichaelRajaguru, GulasekaranEyupoglu, CanRak, EwaFaes, LucaRalston, John P.Fahs, MarwanRamirez, Pedro E.Fakotakis, NikosRamírez-Argáez, MarcoFang, ChangmingRamirez-Rojas, AlejandroFang, Chin YiRamos, HiginioFang, Shih-HauRamos, Pedro L.Farantos, Stavros C.Ramstead, Maxwell J. D.Fattal, EyalRana, PratipFavretti, MarcoRandall, Sean M.Fawzy, AbdelhameedRao, Raveendra K.Febres, GerardoRaptopoulos, Christoforos L.Febvre, PascalRashad, Ahmed M.Fedorov, Vladimir E.Rashel, MasudFeinman, Richard D.Rashid, MuhammadFeleagă, LilianaRastelli, GianlucaFelice, DomenicoRau, A. R. P.Felipe, José LuisRaudino, AntonioFeng, RuiRavber, MihaFernandes, Carlos M.Raykova, RenetaFernandes, Duarte Manuel AzevedoRazeghi, BehroozFernandez, AlbertoRazi, AdeelFernández, Francisco JavierReboiro, MartaFernandez, Luis ManuelRebolledo, RolandoFernández-Alcalá, Rosa M.Redner, SidneyFernandez-Veiga, ManuelRegulski, KrzysztofFerraro, DarioRehman, SaadFerreira, ManuelReiber, Johan H. C.Ferreira, Marta Susana RibeiroReittu, HannuFerreira, PauloRen, JiandongFetaji, BekimRen, JianfengFicco, MassimoResca, Lorenzo G.Ficheux, QuentinRestuccia, LilianaFields, ChrisReygner, JulienFilho, Mauro FazionReynolds, KeithFilippov, SergeyRhi, Seok-HoFiori, SimoneRiascos, Alejandro PérezFirpo, Marie ChristineRiaz, QaiserFisicaro, EmiliaRibeiro Gabriel, Paulo HenriqueFivos, PapadimitriouRibeiro, FabianoFlores-Márquez, E. LeticiaRibeiro, Raphael F.Flórez-Orrego, DanielRice, MichaelFlorio, GiuseppeRichardson, MichaelFloros, ChristosRigby, JohnFodorean, DanielRighi, MarcoFokoué, ErnestRighi, RodrigoFondón, IreneRighi, SimoneFonnesbeck, ChristopherRincon, AngelFonollosa, Javier RodríguezRios-Figueroa, Homero VladimirFontalvo, ArmandoRistic, MiroslavFontana, ClaudioRivera, GilbertoFont-Clos, FrancescRizzuto, LuciaFontes, RamonRobb, GordonForero, Jorge DuarteRobert, NewcombForestiero, AgostinoRocco, DavideFortin, Jean-YvesRocha, J. LeonelFortuna, LuigiRodrigues, AlexandreFoschini, LuigiRodrigues, PauloFossion, RubenRodríguez, FranciscoFournier, BertrandRodríguez, JavierFournier, SébastienRodriguez, NibaldoFrąckiewicz, PiotrRodriguez-Lopez, PabloFragoulis, GeorgeRogovoy, Anatoly A.Francoise, Jean-PierreRogowski, KrzysztofFrangopoulos, ChristosRoh, HeejunFränti, PasiRoig, Ignacio BoschFrei, ChristophRojo, Maria AngelesFreij-Hollanti, RagnarRoland, MichaelFrery, AlejandroRomán, Pablo E.Freund, JanRoman, Raul-CristianFriedenberg, JayRomano, VittorioFriederich, SimonRomero, GuillermoFriedman, YaakovRomero, GustavoFriedrich, PatrickRomero-Rochin, VictorFritschek, RickRosano, MariangelaFrizzo, Clarissa P.Rosas, FernandoFrolkova, AnastasiaRosenberger, DavidFrolov, AlexeyRossi, RiccardoFrydryszak, AndrzejRossomando, FranciscoFu, ShuangshuangRosu, HaretFuchs, HansRotaru, AndreiFuh, Chiou-ShannRoterman, IrenaFujimto, ShoujiRothkrantz, LeonFukumitsu, MasayukiRoulia, MariaFülöp, BazsóRoversi, José A.Furmańczyk, KonradRowland, ZuzanaFuruhata, HitoschiRoy, ArkoFusek, MichalRubaszek, MichałFuso, FrancescoRubega, MariaGabbana, AlessandroRubi, Miguel J.Gabrielli, AndreaRubin, SergioGadomski, AdamRuf, AlexanderGagliardi, GianfrancoRundo, LeonardoGaida, DanielRuppert, LászlóGajic, DragoljubRusowicz, ArturGalam, SergeRusso, MicheleGallerati, AntonioRusyn, BohdanGallo, DiegoRyabko, BorisGallo, Waldyr Luiz RibeiroRybin, PavelGallos, LazarosRydzewski, JakubGalván-Tejada, Carlos E.Rza̧dkowski, GrzegorzGalvis-Carreno, LauraSaad, DavidGalyaev, AndreySaadane, RachidGameiro, AtilioSaavedra, GabrielGammaitoni, LucaSabag, OronGan, MinSabín, CarlosGao, ChangjunSachdeva, RashiGao, CongSachlas, AthanasiosGao, JianSadovskiǐ, Michael V.Gao, ShangceSaeed, KhalidGarcia de Andrade, LuizSaeed, NaghamGarcía Márquez, Fausto PedroSafron, AdamGarcía Salvador, RosaSagar, Robin P.García, Jesus EnriqueSaha, SangeetGarcia, MagdalenaSahgal, PranshuGarcía, Pedro GonzálezSahin, Ahmet Z.García, Reinaldo GarcíaSahut, Jean-MichelGarcia, RogérioSaia, RobertoGarcía-Frias, JavierSaigo, HayatoGarcía-Gil, DiegoSaito, KazuyaGarcia-Hernandez, Jose JuanSakan, NenadGarcia-Herrero, FranciscoSakkopoulos, EvangelosGarcía-López, Juan HugoSakowski, PawełGarcía-Loygorri, Juan MorenoSalahdine, FatimaGarcía-Martínez, BeatrizSalazar, AddissonGarcía-Medina, AndrésSalimi, MehdiGarcia-Pacheco, Francisco JavierSalina, GaetanoGargano, FrancescoSalomon, MichelGargiulo, Gaetano D.Salomon, PeterGastpar, Michael C.Samakovitis, GeorgiosGatto, Bernardo B.Samarov, Artemiy A.Gautier, RomainSampaio, MarcosGavanelli, MarcoSamuel, JohnGavrilov, MomčiloSánchez-Cañizares, JavierGe, HaoSanchez-Soto, LuisGeier, MartinSanei, SaeidGeiger, Bernhard C.Sang, YingpengGençağa, DenizSantamato, AlbertoGeng, YanlinSantos, Alan Costa DosGenovese, MarcoSantos, JoséGeorgiev, Danko D.Santos, José Joaquim ConceiçãoGeorgiev, GeorgiSantos, Nilton O.Georgieva, AtanaskaSantos, RicardoGervino, Gianpiero P.Santos-García, GustavoGessner, ManuelSantucci, EnricaGewen, YiSanz Ortiz, Ángel SantiagoGherardini, FrancescoSanz, Ángel S.Ghoreishi, Seyede FatemehSanz, RicardoGhosh, AritraSaracco, FabioGiannakis, KonstantinosSarasa-Cabezuelo, AntonioGiannerini, SimoneSaridakis, EmmanuelGiarré, LauraSarjas, AndrejGibbons, GarySarkar, DebajyotiGibson, Jerry D.Sarkar, Nurul I.Giffard-Roisin, SophieSarmento, HugoGiffin, AdomSarris, IoannisGigante, GabriellaSason, IgalGilja, GordonSassetti, MauraGil-Villegas, AlejandroSato, IsseiGiorgi, Gian LucaSaucan, EmilGisin, NicolasSauer, NathalieGitis, ValeriSavel′Ev, SergeyGiudici, PaoloSawerwain, MarekGiuliani, AlessandroSbert, MateuGiuliano, FabrizioSburlea, Andreea IoanaGiurcaneanu, Ciprian DoruScaramozzino, PasqualeGiusteri, Giulio GiuseppeSchclar, AlonGlattli, ChristianSchinckus, ChristopheGlavatskiy, KirillSchindelhauer, ChristianGlowacz, AdamSchindler, JosephGnatyuk, SergiySchlitter, JürgenGnjatović, MilanSchmelzer, Jürn W. P.Godec, AljazSchmidt, Eric A.Godoy, DanielaSchmidt, HelmutGoldbaum, DavidSchmitt, MichaelGoldstein, MosheSchnurr, AlexanderGoltsev, AlexanderScholz, MichaelGomes Garcia, AdrianoSchreiber, AndreasGómez, Diego Sáez-ChillónSchrinski, BjörnGómez, HelenaSchuh, AndreasGomez, PabloSchulman, LawrenceGomez, SergioSchuster, SebastianGómez-Aguilar, José FranciscoSchwenker, FriedhelmGómez-Valent, AdriàScott, David W.Gonçalves, André R.Scott, RobertGonçalves, HernâniSebastian, Jucu IoanGonzález Manzano, LorenaSebastiani, LorenzoGonzález, HugoSedrakian, ArmenGonzález-Gaxiola, OswaldoSee, Chan HwangGonzalez-Marcos, AnaSeely, Andrew J. E.Gonzalez-Marin, AdelaSelesnick, IvanGonzález-Pachón, JacintoSellami, AkremGonzalez-Parra, GilbertoSemieniuk, GregorGoodarzi, MarjanSempi, CarloGorban, Alexander N.Sen, PinarGórecki, TomaszSenno, Gabriel IgnacioGorska, KatarzynaSeo, HwajeongGosak, MarkoSeo, HyunseokGough, JohnSequeira, JeanGoupil, AlbanSerackis, ArturasGouveia, SoniaSerban, FlorentinGovedarica, MiroŞerban, GheorgheGradenigo, GiacomoSergi, AlessandroGrądzki, RafałSergi, Pier NicolaGrahovac, DanijelSerota, RostislavGranville, LisandroServa, FedericoGravner, JankoServadio, Joseph L.Gray, ChrisServedio, Vito D. P.Grechuk, BogdanSesma-Sara, MikelGrigg, NeilSestras, PaulGrigoras, VictorSfetcu, Razvan-CornelGrigoryan, ArtyomShafiq, AnumGrimaudo, RobertoShagrir, OronGryzlova, Elena V.Shalyt-Margolin, Alexander E.Grzegorzewska, EmiliaSharapova, PolinaGu, BingSharma, VikasGualtieri, CarloSharov, German SergeevichGuariglia, EmanuelShemyakin, ArkadyGubitosa, JenniferShen, Chien-wenGuedes, EveraldoShen, CongGuedes, Maria AlexandraSherman, EilonGuerreiro, Gracinda RitaSheu, Ruey-KaiGuerrero, Juan I.Shi, LeiGuglielmetti, FabriziaShieh, Chin-ShiuhGuha, ParthaShihavuddin, ASMGuido, Rodrigo CapobiancoShikhovtsev, ArtemGuillén i Fàbregas, AlbertShin, DongheeGulácsi, ZsoltShirazi, AbolfazlGulgowski, JacekShishlenin, Maxim A.Gülich, Johann FriedrichShlesinger, Michael F.Gunasekaran, NallappanShmueli, ErezGünlü, OnurSiami-Namini, SimaGuo, YanhuiSicuro, GabrieleGupta, Daya ShankarSiczek, KrzysztofGupta, DeepakSidorov, DenisGupta, SukritSidorov, Nikolay A.Gutub, AdnanSiebenhühner, FelixGuzzi, FrancescoSiekmann, IvoGwak, BogeunSigalotti, Leonardo Di G.Ha, Dac-BinhSik-Lanyi, CeciliaHabib, UsmanSilva, ArlindoHackley, Steven A.Silva, Luiz Eduardo VirgilioHadar, OferSimani, SilvioHadjesfandiari, Ali RezaSimeonov, IvanHaeusler, EdwardSimionescu, MihaelaHagara, MartinSimm, NickHaghbayan, MohammadSimons, GregoryHagiwara, ManabuSingh, AmarHajiyev, Natig Qadim-OgluSingh, KuldeepHaken, HermannSingh, PrameshHalanay, AndreiSingh, ShellyHall Barbosa, Carlos RobertoSingharoy, AbhishekHamada, MohamedSiqueira, Hugo ValadaresHamann, HeikoSirbu, Ioana-GabrielaHamdache, MohamedSirmans, G. StacyHammad, Mohamed AdelSitar-Tăut, Dan-AndreiHammoudeh, MohammadSiuta-Olcha, AlicjaHamza, RafikSivanandam, SivasankaranHand, Paul E.Siwik, LeszekHangan, AncaSkaržauskienė, AelitaHannig, JanSkiba, MartaHannisdal, BjarteSkilling, JohnHansen, Lee D.Skoczeń, AndrzejHansen, NielsSkordas, EfthimiosHanslmeier, ArnoldSładkowski, JanHariri-Ardebili, Mohammad AminŚlepaczuk, RobertHarremoës, PeterSlodek, AnetaHartley, IanSlyne, FrankHartmann, CarstenSmieszek, MiroslawHasimoto-Beltran, RogelioSmirnov, Ivan V.Haspel, NuritSmith, AaronHassan, Mohamed AhmedSmith, D. HudsonHategan, Camelia DanielaSmolinski, Kordian AndrzejHatemi-J, AbdulnasserSmotrovs, JurisHatzilygeroudis, IoannisSmyk, EmilHaun, AndrewSokolovska, NataliyaHausenblas, ErikaSokolovski, DmitriHaven, EmmanuelSolà, JoanHawkins, RaymondSolanes, AleixHayashi, MasahitoSolis-Prosser, Miguel AngelHe, JiguangSonderegger, DerekHealey, RichardSone, AkiraHéam, Pierre-CyrilleSonnino, GiorgioHegedűs, FerencŠorli, AmritHeitmar, RebekkaSotiriadis, PaulHelbig, MardeSoualhi, AbdenourHelle, MikkoSouto, AndréHemanth, JudeSouza, Richard DemoHerber, Daniel R.Spanakis, Emmanouil G.Hernández, FreddySparavigna, Amelia CarolinaHernández-Cruz, CésarSperandio, MauricioHernández-Fernández, AntoniSperlì, GiancarloHernández-Pereira, ElenaSridhar, KaushikHerrera Corral, Gerardo A.Srivastva, DeepikaHerrera-Tapia, JorgeStabile, GiovanniHerrmann-Pillath, CarstenStanciu, CristianHess, KarlStanislavsky, AleksanderHess, KathrynStankovic, RadomirHeszberger, ZalánStarikovskiy, AndreyHildebrandt, AndreStaudte, RobertHill, HolgerStefanatos, DionisisHingerl, KurtSteinmetz, Peter N.Hirata, YoshitoStel, HenriqueHirche, ChristophStepaniuk, JarosławHirose, YuichiStepien, MariuszHirota, OsamuStern, JulioHirsch, Jorge GustavoStern, SebastianHladek, DanielStoean, CatalinHlavackova-Schindler, KaterinaStoianov, Ivilin PeevHnilo, AlejandroStornaiolo, CosimoHo, NhatStoyanov, BorislavHoblos, GhalebStramaglia, SebastianoHoey, JesseStrbak, OliverHoffmann, Karl HeinzStrečka, JozefHoffmann, MarkusStrelchuk, SergiiHolevo, AlexanderStrunk, ChristophHolik, FedericoStübinger, JohannesHolzmann, MarkusStudenka, BreannaHong, DanfengŠtula, MajaHong, Jang-EuiStutzer, MichaelHönnicke, Marcelo GonçalvesSu, Bo-chiuanHorchani, RidhaSubasi, AbdulhamitHorla, DariuszSuchecki, KrzysztofHorn, BartSuciu, AlinHorng, Ji-HweiSuciu, GeorgeHoroshko, DmitriSujit, SheebaHorslen, BrianSujit, Sheeba JeniferHorvatić, DavorSun, JiandeHorwitz, LawrenceSun, JieHotza, DachamirSundar, BhuvaneshHoughton, Conor J.Sunderland, CarolineHristina, KulinaSung, JungyeonHristopulos, Dionissios T.Surowka, Artur D.Hristova, SnezhanaSutor, AlexanderHrymak, Andrew NickSuzić, SinišaHsin, Kun-YiSuzuki, HiroyukiHsu, Li-YiŚwiercz, MirosławHsu, Ming-FuSyed, Mujahid N.Hu, DongdongSyriopoulos, KonstantinosHu, RuifengSzałowski, KarolHu, YuSzavits-Nossan, JurajHua, XiaoqiangSzczepanek, RobertHuang, GuangweiSzczepanski, JanuszHuerta Cuellar, GuillermoSziebig, GaborHulea, MirceaSzopa, MarekHung, Jui-ChengSzymański, GrzegorzHussain, IqtadarSzymoniak, SabinaHussein, MoustafaTabrikian, JosephHuveneers, FrançoisTabus, IoanHwang, Jan-PanTaddei, FabioIana, Vasile-GabrielTaj, DavidIancu, BogdanTakabe, SatoshiIannazzo, DanielaTakahashi, FutoshiIannuzzi, ClaraTakasawa, KenIbanez-Molina, Antonio J.Talleda, Pere VilaIbrahim, MohamedTam, Ka MingIchiba, TomoyukiTama, Bayu AdhiIdrisov, EdvinTamascelli, DarioIeracitano, CosimoTamm, MartinIglesias-Parro, SergioTan, BingyaoIguchi, HideoTanaka, HitoshiIhle, AndreasTaneco-Hernández, Marco AntonioIkeda, KazukiTanveer, SheikIkegami, TakashiTapias, DiegoIlyas, SadiaTarel, Jean-PhilippeImran, JamalTaskinen, SaraIngrid, TonhajzerováTawose, Olamide TimothyIoan, BatranceaTayyab, MuhammadIoan, TudosaTchangani, AyeleyIoana, AdrianTchier, FairouzIomin, AlexanderTchórzewska-Cieślak, BarbaraIp, Ryan H. L.Teh, Kah ChanIria, JoseTeljeur, ConorIsar, AurelianTerebes, RomulusIslam, MD SaifulTessarotto, MassimoIslam, RabiulThakar, Parth S.Ismail, MohamedThingna, JuzarIsokawa, TeijiroThomsen, KnudIssa, IbrahimThyagaturu, AkhileshIsvoranu, DragosTian, JingIvan, PiresTian, LiangIvanis, PredragTibiriça, Cristiano BigonhaIvanov, Martin V.Tipantuña, ChristianIvanov, OvidiuTittarelli, AndreaIvanov, Peter AleksandrovTiwari, PrayagIvanov, SergeiTkachenko, PavloIzonin, IvanTlelo-Cuautle, EstebanIzydorczyk, JacekTobochnik, JanJacak, JanuszTohme, FernandoJacobs, DonaldToker, OnurJaeger, GreggToldinas, JevgenijusJafari, SaeidToledo, Benjamin A.Jahn, MalteToma, AidaJajcay, NikolaToma, Sorin GeorgeJakimowicz, AleksanderTook, CliveJakóbczyk, LechToranzo, Irene V.Jakubský, VitTorp, OlavJamil, MohsinTorre, Iván G.Jammeh, EmmanuelTorres, AbelJamro, ErnestTorres-Arenas, JoséJanno, JaanTóth, GézaJäntschi, LorentzTraer, JamesJarzynski, ChristopherTrakadas, PanagiotisJasinski, MichalTran, Gia KhanhJauregui, MaxTran, HuyJaúregui, RocíoTran, Minh-QuangJaved, FarrukhTrecroci, AthosJekova, IrenaTremeau, AlainJelinek, HerbertTrenchi, LorenzoJen, Chun-PingTriantafyllou, IoannisJenkins, AlejandroTrifina, LucianJennings, Robert C.Trifonov, Peter V.Jeong, KabgyunTrivellato, BarbaraJeong, Young-SeobTrujillo, Logan T.Jepps, OwenTsai, Chia-WeiJi, WeiqiTsai, Pei-HsuanJiang, LiTsang, HectorJiang, MingzheTsekouras, George E.Jianu, IuliaTseng, Chih-yuanJin, MingluTseng, Yuen-HsienJinwoo, LeeTsiakas, KonstantinosJipa, AlexandruTsimpiris, AlkiviadisJizba, PetrTsipouras, Markos G.Joffily, MateusTsironis, Giorgos P.Jones, MylesTsoumakos, DimitriosJones, Rodney P.Tsung, Chen-KunJordan, StephenTsurutani, Bruce T.Jordanova, PavlinaTsutsui, IzumiJose, Sharu TheresaTuegeh, MaickelJoshi, Gyanendra P.Tull, SeanJoslin, ChrisTunc, CemilJung, MinchaeTweneboah, OseiJung, Se HoonTzanetos, AlexandrosJupowicz-Ginalska, AnnaUddin, JiaJuretić, DavorUeltzhoeffer, KaiJuszczuk, PrzemysławUffink, JosJůza, JosefUgarte, OrlandoKaabar, Mohammed K. A.Ul Haq, Muhammad AzizKaandorp, JaapUllah, KifayatKabir, GolamUmlauf, NikolausKadovic, RatkoUngureanu, FlorinaKadry, SeifedineUnhelkar, BhuvanKainen, PaulUttam, ShikharKaiser, M. ShamimVadászné Bognár, GabriellaKalenik, MarekValdés-Hernández, AndreaKałuża, TomaszValverde-Albacete, Francisco J.Kamberaj, HiqmetValyrakis, ManousosKamola, MariuszVardasca, RicardoKanamori, TakafumiVarga, DomonkosKang, Dong-KiVarotsos, PanayiotisKania, KrzysztofVasco-Calle, Diego AndrésKaniadakis, GiorgioVasilaki, EleniKanniainen, JuhoVasoya, ManishKantartzis, Nikolaos V.Vavrek, RomanKanwal, NadiaVázquez Polo, Francisco JoséKapcia, Konrad JerzyVega, MiguelKarabasevic, DarjanVeiga-Suárez, FernandoKaragrigoriou, AlexVenkitasubramaniam, ParvKarakatsanis, LeonidasVeranic, PeterKaram, AhmedVerde, FrancescoKarampidis, KonstantinosVerdoolaege, GeertKarczmarczyk, ArturVeress, ÁrpádKarczmarek, PawełVergara, ChristianKari, LeifVergara, DiegoKarimi, NaderVerma, RajkumarKarimullin, KamilVersaci, MarioKarpinski, MikołajVesin, Jean-MarcKarsznia, Krzysztof R.Vessio, GennaroKarthick, S. K.Vetschera, RudolfKarwasz, GrzegorzVicente, RenatoKatayama, ShotaVicente, Rodriguez MontequinKateris, DimitriosVidal, Jorge MaestreKatona, JozsefVidal-Tomás, DavidKavic, MichaelVieru, DumitruKawaguchi, YukiVillarroel, JavierKawamoto, ShoichiVillaseñor, Eduardo J. S.Kay, BernardVillecco, FrancescoKay, JamesVilone, DanieleKay, Jim W.Viňáš, JánKeeler, CynthiaVinck-Posada, HerbertKelemen, MichalVinogradova, IrinaKeler, AndreasVirgala, IvanKeller, KarstenVirgo, NathanielKelso, J. A. ScottVisconti, PaoloKenig, BatyaVisintin, MonicaKenna, RalphVisser, ManusKenyeres, MartinVitázek, IvanKeppens, ArnoVlădeanu, CălinKertész, JánosVlaj, DamjanKeshmiri, SoheilVogel, Eugenio E.Keziou, AmorVoicu, CarmenKhachay, MichaelVolchenkov, DimitriKhalique, ChaudryVolosencu, ConstantinKhan, AfrasayabVon Renesse, Max KonstantinKhan, Muhammad HassanVopson, Melvin M.Khan, ShahidVora, AnkitKhater, Mostafa M. A.Vujović, IgorKhlopov, MaximVulpiani, AngeloKhudaBukhsh, AshiqueVyas, RiteshKhvedelidze, ArsenWacławczyk, MartaKiang, Jean-FuWaegell, MordecaiKiefer, AlexWaller, Lance A.Kim, Byung-GyuWan, KaiKim, Byung-HoonWang, HongshengKim, Chang SubWang, Jian-ShengKim, CheonshikWang, JihengKim, Dong-HeeWang, LeleKim, Eun-jinWang, LiangliangKim, HwajoonWang, Lin-ShuKim, Hyun CheolWang, PingKim, Hyun JinWarren, Patrick B.Kim, IlkiWashington, Lawrence C.Kim, JeonggukWasiak, AndrzejKim, Jong-ChanWatanabe, EiichiKim, Jong-MinWebber, Robert J.Kim, Jong-MyonWedrowska, EwaKim, JoonhoWei, HaoranKim, Jung TaeWeinberger, NirKim, Kyung SooWeinert, FriedelKim, Man-HoeWeinfurter, HaraldKim, MoonseongWeiss, MatthiasKim, Na YoungWeitschek, EmanuelKim, SooyoungWen, Chih-YuKinney, DavidWen, JianmingKinouchi, OsameWeng, Jian-JiaKirmani, Syed N. U. A.Werman, MichaelKirwan, Albert, Jr.Weron, RafałKizek, JánWettstein, MartinKizilersu, AyseWharton, KenKlages, RainerWhite, JeffreyKlein, IngoWibowo, SantosoKleinschmidt, JoãoWibral, MichaelKlimek, ChristianWichert, AndreasKlimov, AndreiWielechowski, MichalKnyaz, VladimirWiesner, KarolineKnypiński, ŁukaszWijayanto, Arie WahyuKocharyan, Armen A.Wijayatunga, PriyanthaKóczy, László Á.Wilkinson, MichaelKoczyk, GrzegorzWinfree, KyleKogias, DimitrisWiseman, Howard M.Kokoulina, ElenaWitczak, MarcinKołaczek, GrzegorzWohlschläger, AfraKolchinsky, ArtemyWojnakowski, MarcinKolomvatsos, KostasWojnicki, MarekKominis, IannisWollstadt, PatriciaKomiyama, JunpeiWollweber, MerveKomsiyska, LidiyaWołosz, Krzysztof J.Kong, LinglongWong, Albert S. Y.Kongar, ElifWoo, Jonh HunKonieczny, PiotrWu, An-YeuKönig, JürgenWu, JianzhongKononovicius, AleksejusWyffels, FrancisKonstantinides, DimitriosWysocki, MarianKontogiannis, IoannisWysokinski, Karol I.Kontogiannis, SotiriosXavier, AlencarKonys, AgnieszkaXiao, DongliangKoppitz, JörgXiao, LiangliangKorbel, JanXie, ShengkunKorkmaz, Mustafa ÇağatayXing, LiKorniejenko, KingaXing, SiyuanKosloff, RonnieXu, HongmingKošnik, NejcXu, KaiKostek, BożenaXu, LangKostic, MilivojeXu, WeihuaKostopoulos, GeorgiosYadav, RajeevKostrzewski, MariuszYamashita, DaichiKot, Alex C.Yan, BinKotropoulos, ConstantineYang, Chun-HaoKotsiantis, SotirisYang, Chun-WeiKou, GangYang, JanghoonKoutlis, ChristosYang, Shanchieh JayKovâcs, PéterYanofski, NosonKovács, RóbertYao, FengKovalsky, Marcelo G.Yarovaya, LarisaKowalewska-Kudłaszyk, AnnaYeh, Sheng LihKozak, JanYngvason, JakobKozielski, MichałYoon, Ki JungKoźlak, JarosławYoshino, KohzohKozyrev, DmitryYoung, AlexanderKrajzewicz, DanielYousefi, BardiaKralj, SamoYoussef Moussa, Miled HassanKramarz, MarzenaYu, Chih-ChangKrapf, DiegoYu, LiliKrasnytska, MarianaYuan, RuoshiKrasowska, MonikaYun, Ji-HoonKratzke, NaneYunusa-Kaltungo, AkiluKrause, DécioYurtkan, KamilKraut, ManfredZabrodin, EvgenyKravchenko, Oleg V.Zafar, AzharKrippendorff, KlausZaidi, AbdellatifKröker, IljaZak, MarekKról, AleksanderZakrzewski, JakubKrpan, LjudevitŽalik, Krista RizmanKrüger, BjörnZambrano-Serrano, ErnestoKryzhanovsky, BorisZanoni, SimoneKrzaczek, PawełZavoli, AlessandroKubiznak, DavidZdravevski, EftimKuchař, JaroslavZeller, Camila BorelliKudryashov, BorisZemouri, RyadKudryavtsev, KonstantinZenil, HectorKułakowski, KonradZhang, GuangmingKułakowski, KrzysztofZhang, GuofengKulikovskikh, IlonaZhang, HengjieKullie, OssamaZhang, JizeKumar, ApurvZhang, JunhuanKumbhakar, ManotoshZhang, SixinKundu, ChinmoyZhao, PanKunert-Graf, James M.Zhbanov, AlexanderKunimi, MasayaZheng, DongdongKunnev, DimiterZherlitsyn, DmytroKuo, Jian-LongZhou, ShengKuok, Sin-ChiZhu, KeKupczynski, MarianZhuo, JiachenKuptsov, MichailZiaeepour, HouriKurubanjerdjit, NilubonZielosko, BeataKuś, MarekZilhao, MiguelKustova, ElenaZinovyev, AndreiKutner, RyszardZivieri, RobertoKuzemsky, AlexanderZloshchastiev, Konstantin G.Kuznetsov, AlexandrZolotukhin, MikhailKvalseth, TaraldZrelli, AmiraKvasov, Dmitri E.Zubkov, MikhailKwek, Leong ChuanZucchelli, GiuseppeKwiecien, JoannaZueco, DavidKwon, HyunZukowski, WitoldKwon, Roy H.Zullo, FedericoKycia, RadosławZunino, Luciano

